# Copy number gain of pro-inflammatory genes in patients with HBV-related acute-on-chronic liver failure

**DOI:** 10.1186/s12920-020-00835-5

**Published:** 2020-12-01

**Authors:** Fengming Sun, Wenting Tan, Yunjie Dan, Xiuhua Wang, Yanzhi Guo, Guohong Deng

**Affiliations:** 1 Department of Infectious Diseases, Southwest Hospital, Third Military Medical University (Army Medical University), Shapingba District, Chongqing, 400038 China; 2 Chongqing Key Laboratory for Research of Infectious Diseases, Shapingba District, Chongqing, 400038 China

**Keywords:** Copy number variations, HBV-related ACLF, GWAS, HBV-ACLF, Acute-on-chronic liver failure

## Abstract

**Background:**

Host genetic factors such as single nucleotide variations may play a crucial role in the onset and progression of HBV-related acute-on-chronic liver failure (ACLF). However, the underlying genomic copy number variations (CNVs) involved in the pathology are currently unclear.

**Methods:**

We genotyped two cohorts with 389 HBV-related ACLF patients and 391 asymptomatic HBV carriers (AsCs), and then carried out CNV-based global burden analysis and a genome-wide association study (GWAS).

**Results:**

For 1874 rare CNVs, HBV-related ACLF patients exhibited a high burden of deletion segments with a size of 100–200 kb (*P* value = 0.04), and the related genes were significantly enriched in leukocyte transendothelial migration pathway (*P* value = 4.68 × 10^–3^). For 352 common CNVs, GWAS predicted 17 significant association signals, and the peak one was a duplication segment located on 1p36.13 (~ 38 Kb, *P* value = 1.99 × 10^–4^, OR = 2.66). The associated CNVs resulted in more copy number of pro-inflammatory genes (MST1L, DEFB, and HCG4B) in HBV-related ACLF patients than in AsC controls.

**Conclusions:**

Our results suggested that the impact of host CNV on HBV-related ACLF may be through decreasing natural immunity and enhancing host inflammatory response during HBV infection. The findings highlighted the potential importance of gene dosage on excessive hepatic inflammation of this disease.

## Background

Hepatitis B Virus (HBV) infection is regarded as a global health issue. The global chronic HBV infection rate in 2015 was estimated at 3.5% involving 257 million people, of which 15–25% died of HBV-related cirrhosis or liver cancer [1]. Caused by severe acute exacerbation of chronic hepatitis B (CHB), HBV-related acute-on-chronic liver failure (ACLF) is a severe life-threatening disease that exhibits a high 28-day mortality rate of more than 15% [2]. Several risk factors have been suggested to be involved in this common complex disease, including hereditary factors, host characteristics, viral factors, and vigorous immune responses [3–5]. However, the pathological processes are still poorly understood.

Host genetic factors likely play a crucial role in the pathogenesis of HBV-related ACLF. Our group has recently performed a SNP-based genome-wide association study (GWAS) of this disease, and identified a highly associated variant rs3129859*C [6]. The related single nucleotide variation is located in human leukocyte antigen (HLA)-DR region and likely participates in the function of the HLA-II-restricted CD4+ T-cell pathway [6]. Previous results deepen our understanding of HBV-related ACLF and confirm the importance of host genetic factors in the pathogenesis of the disease. In addition to SNPs, copy number variations (CNVs) as another main type of genetic variations also exhibit a high diversity in human population and are associated with many human diseases [7]. For example, susceptible CNVs on chromosome 1p36.33 [8] and 15q13.3 [9] were proved to be highly correlated with HBV-related hepatocellular carcinoma (HCC), and CNVs on chromosome 5q35.3 [10] may potentially affect HBV infection by integrating the HBV P gene into natural killer cells. However, direct evidences of association between host CNVs and HBV-related ACLF remains unknown.

In this study, we used Affymetrix Genome-wide Human SNP Array 6.0 to identify high-quality CNV genotyping data for HBV-related ACLF in a Chinese population, and then performed a CNV-based GWAS aiming to expand the scope of genetic screening and further study the underlying genetic and molecular mechanism of HBV-related ACLF.

## Methods

### Participants, CNV detection and quality control

**Fig. 1:**
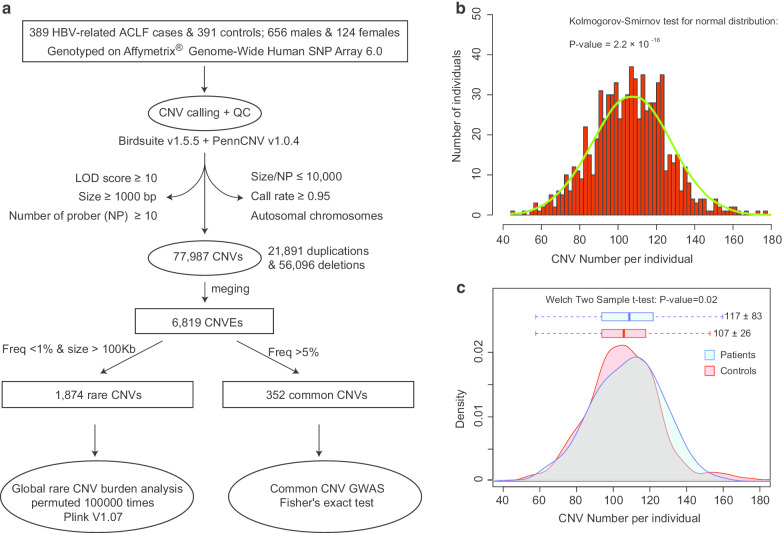
Overall analysis methods and genotyping results. **a** Overall flow chart of genotyping and analysis. Some intermediate results and final results were marked in the figure. The normal distribution test was applied to verify the randomness of the results (**b**), and the distribution of the CNV number of each individual was very close to normal (*P* = 2.2 × 10^–16^). However, the distribution showed significant difference between HBV-related ACLF cases and AsC controls (**c**), indicating the potential host CNVs difference of the HBV-related ACLF

A Chinese population with 780 qualified participants was screened from our previous study (population group of “GWAS stage”), including 389 patients with HBV-related acute-on-chronic liver failure (ACLF, cases) and 391 asymptomatic HBV carriers (AsCs, controls) (Additional file 1 and Additional file 2) [6]. The detailed diagnostic criteria for HBV-related ACLF and AsC were also described previously [6]. Standard procedures were conducted to extract the genomic DNAs from leukocytes in peripheral blood. Raw signals of copy number variations (CNVs) were detected using the Affymetrix genome-wide human SNP array 6.0 (Affymetrix, Santa Clara, California, USA), and the CNV genotypes were further determined plate-by-plate using Birdseye (version 1.5.5) under the JCH model (Japanese and Chinese model) [11]. High quality CNV calls should meet five criteria [12]: logarithm of odds (LOD) score ≥ 10, Size ≥ 1000 bp, the number of probes per CNV (NP) ≥ 10, Size/NP ≤ 10,000, and call rate ≥ 0.95. In addition to Birdseye, the software PennCNV (version 1.0.4) [13] was also applied as a complementary algorithm to determine CNVs using the default parameters (Fig. 1a). For the distribution of CNV numbers per sample, Kolmogorov–Smirnov test was used to measure the goodness of fit for normal distribution, which was accomplished by R scripts.

### Determination of common and rare CNVs

A CNV event (CNVE) was clustered from a series of CNV calls that descended from a common ancestral mutation event [12] and had a pair-wise reciprocal overlapping rate of over 50%. Contributing CNV calls of a CNVE may have slightly different breakpoints in the genome. The physical extent of the CNVE was defined as the minimum region covering over 90% of these related CNVs. Meanwhile, the carrier frequency of a CNVE was defined as the proportion of individuals that carried the contributing CNV calls. The common and rare CNVs were CNVEs that have frequency values of > 5% and < 1%, respectively. Additionally, rare CNVs were further filtered by the genome size of > 100 kb. Genes within CNVEs were extracted based on the annotation file from the UCSC Genome Browser (version NCBI36/hg18). All of the above steps were implemented in our in-house Perl scripts.

### Global burden and genome-wide association study

Plink-1.07 [14] was used to test whether HBV-related ACLF patients exhibit a greater burden of rare CNVs relative to AsC controls. Statistical significance was established through 10,000 times of permutation. All rare CNVs were divided into three groups according to the genomic size, and each size interval was further divided into three types: duplication, deletion and the combination of them. In addition, two aspects were considered during the detection, including the number of segments per person (RATE) and the proportion of sample with one or more segments (PROP). *P* values < 0.05 were considered statistically significant. Fisher's exact test was conducted to perform genome-wide association analysis for both common duplication and deletion CNVs. The odds ratios (ORs) were calculated from the formula of (n_A_ × m_a_)/(n_a_ × m_A_), where n_A_ and m_A_ are the total numbers of participants who carry the target CNVs in cases and controls, respectively. The n_a_ and m_a_ are the total numbers of participants who exhibit normal condition in the detected region or carry the other types of CNVs in cases and controls, respectively.

### Transcriptome analysis and miRNA target prediction

Transcriptome data was collected from NCBI by querying the project number of PRJNA360435, of which 5 ACLF patients (Patient1-T0 to Patient5-T0) and 4 healthy individuals (Control1-T0 to Control4-T0) were selected for further analysis [15]. Raw RNA sequencing data from purified CD14+ monocytes was downloaded, which was sequenced by Illumina NextSeq 500 with a single end of 75 bp. In order to calculate gene expression levels, filtered sequencing data was aligned to the UCSC human gene sets (version NCBI36/hg18) using SOAP2 [16]. Only the unique alignment results were considered to generate reads per Kb transcriptome per million mapped reads (RPKM) values that represented for the relative expression level. The RPKM method eliminates the influence of gene size when comparing expression levels between genes. Based on the expression pattern, the Kolmogorov–Smirnov test was applied to filter samples exhibiting large distribution bias among samples. To observe the expression pattern of the HLA-A gene in more patients with HBV-related liver failure, we queried the key words in the Gene Expression Omnibus (GEO) Profiles database from the NCBI. Expression data of 17 HBV-related ALF samples were obtained under an accession number of GDS4387. The potential miRNA binding sites were predicted using MegaBLAST [17] based on the mature sequences downloaded from miRBase [18]. Results with the highest score, located in 3′UTR (protein coding genes), and mapped 2–8 bp at the beginning of mature miRNA were considered for further analysis [19].

### Enrichment of KEGG pathway

Kyoto Encyclopedia of Genes and Genomes (KEGG) pathway enrichment was conducted in three steps. Firstly, all target genes (TGs) were queried against KEGG orthology (KO) in the database (https://www.kegg.jp) to determine the related pathways. Secondly, a hypergeometric test was used to predict KEGG pathways that were significantly enriched in TGs relative to the genomic background of all genes with KEGG annotations. Thirdly, a Bonferroni correction was calculated to control type I error due to multiple comparisons (threshold: corrected *P* ≤ 0.05).

## Results

### CNV detection

For 389 HBV-related ACLF cases and 391 AsC controls, the Birdsuite and PennCNV algorithms yielded 77,987 CNVs (21,891 duplications and 56,096 deletions) in total with a median size of 569,849 bp. All CNV calls were clustered into 6,819 CNVEs, where 4,413 (64.72%) were singletons (Fig. 1a and Additional file 3). The frequency distribution of CNV number per individual (CNPI) was close to the normal distribution (*P* value = 2.2 × 10^–16^, Fig. 1b). Meanwhile, the mean values of CNPI were statistically different between the HBV-related ACLF and the AsC group (*P* value = 0.02, Fig. 1c), which were 117 ± 83 and 106 ± 26 (mean ± SD) respectively. In total, 352 and 1,874 CNVEs were classified as common and rare CNVs (Fig. 1a), respectively, where 331 common CNVs (~ 94%) could overlap (coverage rate > 0.5) with the CNVs from the HapMap database.

### Global burden analysis of rare CNVs

**Fig. 2:**
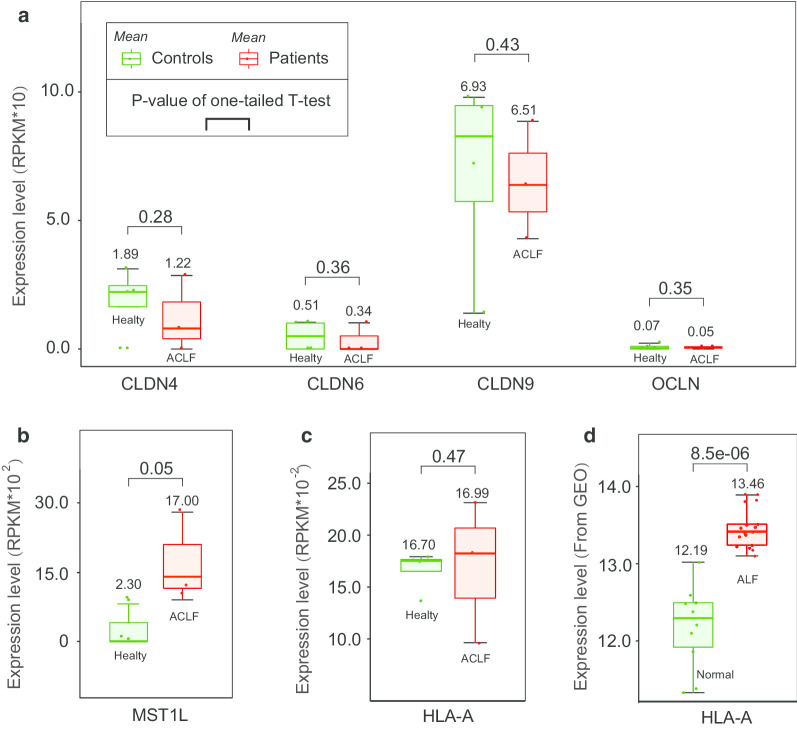
Expression patterns of the potential disease-related genes. Data collection and Expressional calculation was illustrated in the methods part. Low copy number of cell adhesion molecules (CAMs) genes may decrease its expressional level (**a**). More copies of MST1L in HBV-related ACLF likely increase the expressional level (**b**). The mean expression level of HLA-A was both relatively higher in HBV-related ACLF (**c**) and HBV-related ALF (**d**) patients than that of controls

Overall, HBV-related ACLF patients exhibited a significantly higher number of rare CNVs per person than the AsC controls (*P* value = 0.03; Ratio of RATE: 2.78/0.66), but the proportion of samples containing rare CNVs showed no difference between the two groups (*P* value = 0.42; ratio of PROP: 0.29/0.28) (Table 1). In detail, HBV-related ACLF patients revealed a high burden of the deletion segments with the size of 100–200 kb, of which the RATE value was more than 4 times than that of AsC controls (*P* value = 0.04). A total of 1805 genes were contained in the deletion regions (Additional file 4). They are significantly enriched in the leukocyte transendothelial migration pathway (*P* value = 4.68 × 10^–3^). Four major sub-functions are affected, including tail retraction, cell motility, docking structure, and transendothelial migration. Twelve key functional nodes (gene products) lost one or more related gene copies, where the most affected node was cell adhesion molecules (CAMs) (Additional file 5) and its mean expression level was relatively lower in HBV-related patients than the healthy controls (Fig. 2a). In other aspects, there was a higher proportion of patients containing the duplication segments with the size of 100–200 kb (*P* value = 0.02), which covered 172 genes but no KEGG pathway was significantly enriched (Additional file 6).

**Table 1:** Results of global rare CNV burden analysis

Type	Size (Kb)	RATE (case/control)	*P* value RATE^a^	PROP (case/control)	*P* value PROP^b^
Duplications and deletions	100–200	2.05/0.50	0.02	0.25/0.22	0.17
	200–500	0.68/0.14	0.05	0.07/0.10	0.93
	> 500	0.05/0.01	0.08	0.02/0.01	0.21
	All	2.78/0.66	0.03	0.29/0.28	0.42
Deletions	100–200	1.26/0.28	0.04	0.12/0.13	0.61
	200–500	0.49/0.07	0.06	0.05/0.06	0.73
	> 500	0.03/0	–	0.01/0	–
	All	1.78/0.35	0.04	0.15/0.16	0.68
Duplications	100–200	0.79/0.23	0.13	0.16/0.11	0.02
	200–500	0.19/0.07	0.24	0.03/0.05	0.94
	> 500	0.013/0.012	0.62	0.013/0.012	0.62
	All	0.99/0.31	0.14	0.18/0.16	0.16

^a,b^ is the numbers of rare CNV segments per person and the proportion of sample with one or more rare CNV segment, respectively

### Association study of common CNVs

**Fig. 3:**
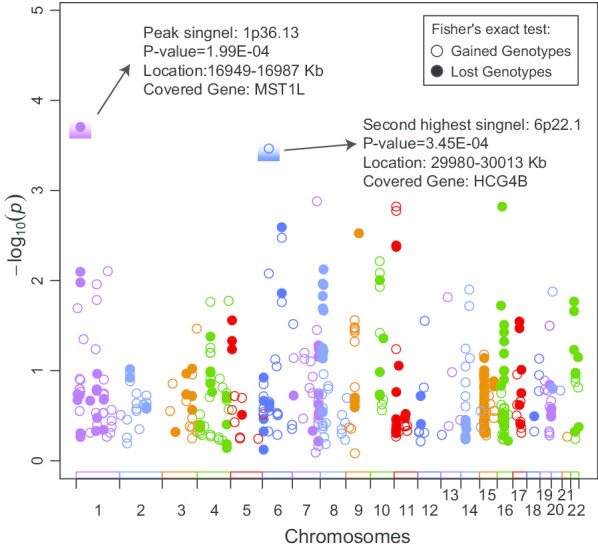
GWAS results of common CNVs for HBV-ACLF. Fisher's exact test was applied to detect all candidate associations for both gained and lost genotypes. The top two highest signals were marked with text description, and the locations were extracted based on the human genome assembly version of NCBI36/hg18

A total of 17 strong disease association signals were detected (Threshold *P* value: ~ 0.01), including 9 duplications and 8 deletions, respectively (Fig. 3 and Table 2). The peak one was a duplicate CNV on chromosome 1 p36.13 (~ 38 Kb, *P* value = 1.99E−04), which had the largest OR value (2.66) among all associates and contained the gene MST1L (macrophage stimulating 1 like). The duplicated CNV was enriched in HBV-related ACLF patients and was associated with greater copies of MST1L compared to the AsC controls, which may further increase its expression level. Transcriptome data showed that the relative expression level of MST1L was significantly higher in HBV-related ACLF patients than in healthy controls (*P* value = 8.20e−4, Fig. 2b).

**Table 2:** Results of association study of common CNVs

Chr	Regions	Type	Foreground (case/control)	Background (case/control)	Odds ratio (OR)	*P* values	Genes included^*^
chr1	16,894,856–16,929,044	Duplication	33/17	337/363	2.09	1.06E−02	
chr1	16,949,054–16,987,299		48/20	307/340	2.66	1.99E−04	MST1L
chr6	103,854,114–103,868,754		79/106	301/274	1.47	1.39E−02	
chr8	7,665,275–7,836,800		142/108	239/258	1.42	1.49E−02	DEFB104A; PRR23D2; SPAG11A; DEFB103A; DEFB105A; DEFB107A; DEFB4A; DEFB106A
chr8	7,202,021–7,259,767		61/38	262/278	1.70	1.09E−02	ZNF705G
chr8	12,260,380–12,487,426		49/28	323/345	1.87	7.56E−03	FAM86B2; FAM66A
chr9	68,055,004–68,312,791		32/15	231/270	2.49	3.01E−03	
chr11	4,928,581–4,930,605		33/57	351/324	1.87	4.25E−03	
chr16	21,297,471–21,501,135		182/147	81/114	1.74	1.52E−03	NPIPB3
chr20	1,509,580–1,520,451	Deletion	303/331	63/42	1.64	1.33E−02	
chr1	173,063,028–173,064,310		87/120	253/230	1.52	7.92E−03	
chr1	110,034,497–110,047,804		89/121	216/196	1.49	1.11E−02	
chr6	29,979,615–30,012,844		49/86	199/171	2.04	3.45E−04	HCG4B
chr6	29,945,293–29,989,326		43/70	121/110	1.79	8.40E−03	HCP5B
chr7	133,446,174–133,449,737		41/71	327/296	1.91	1.33E−03	
chr10	45,905,767–46,573,925		129/97	254/288	1.51	6.14E−03	SYT15; NPY4R; GPRIN2
chr14	40,697,979–40,727,099		47/28	269/289	1.80	1.27E−02	

^*^Genes include functional genes and lncRNA genes

The second-strongest associate was a deletion CNV on chromosome 6 p22.1 (~ 33 Kb, *P* value = 3.45e−04), which was enriched in AsC populations and contained a long non-coding RNA gene HCG4B (HLA complex group 4B). HBV-related ACLF patients tended to contain relatively more copies of HCG4B than AsCs. The mean expression level of HLA-A was higher in HLA-A in HBV-related ACLF or ALF patients than that in healthy controls (Fig. 2c, d). More copies of HCG4B likely resulted in greater gene expression of HLA-A, and the positive correlation may be caused by the competing endogenous RNAs (ceRNA) of lncRNA. A total number of 6 potential sponging microRNAs between HCG4B and 3′UTR of HLA-A were predicted, where miR-6823-5p had the largest prediction score (Additional file 7). Except for two top signals, the 6 remaining associations could also contain gene elements, notably a duplicate CNV on chromosome 8 that covered 7 beta defensin genes (Table 2).

## Discussion

Aiming to explore the risk CNV in HBV-related ACLF, we performed a global burden analysis and a genome-wide association study of 389 HBV-related ACLF cases and 391 AsC controls. A series of high-quality CNVs were identified using SNP array technology, where over 94% of common CNVs overlapped with the HapMap database, providing a strong foundation for subsequent studies. Our results showed that HBV-related ACLF patients tend to contain more short rare CNVs (100–200 Kb) than AsCs, indicating a CNV burden difference between the two groups of patients. Moreover, a total number of 17 common CNVs were found to be significantly associated with HBV-related ACLF. These findings suggested that host genetic copy number variations likely play an important role in disease onset. Further studies implied that genes within related CNVs may participate in decreasing natural immunity and enhancing host inflammatory response during HBV infections.

Compared to AsC controls, HBV-related ACLF population exhibit a higher burden of rare CNVs with the deletion genotype (Table 1), which resulted in a lower copy number of genes related to the leukocyte transendothelial migration pathway (LTMP). The most affected genes are cell adhesion molecules (CAMs) [20], which are the key genes regulating transendothelial migration and play an important role in the firm adhesion of leukocytes during transmembrane transport [21] (Additional file 5). Transcriptome data revealed that low CAM gene copies may further decrease its expression level (Fig. 2a). As one of the important types of leukocytes, natural killer (NK) cells have been found that its peripheral number, cytotoxicity and killing activity were decreased or downregulated in patients with HBV-related ACLF [20]. NK cells are main cellular responders after HBV infection, and the abnormal status can induce severe liver injury [22, 23]. Evidence has shown that NK cells facilitate the cellular immunity of HBV-related ACLF mainly through perforin and granzymes, or interacting with target cell death receptors [22, 24, 25]. Low gene dosage of CAMs may reduce the migration activity of NK cells and further reduce its cellular immunity.

For association studies, the strongest association signal was a duplication segment with a length of ~ 38 Kb, covering only the MST1L (Macrophage stimulating 1 like) gene (Table 2). MST1L is homologous to macrophage stimulating protein (MSP), and its first 6878 bp sequence was 96.1% identical to MSP [26]. MST1L was once thought a pseudogene of MSP due to the frameshift and termination mutations. However, Yoshimura et al. found that MSP homologous genes could express in HepG2 cells [27], suggesting that MST1L may have transcriptional activity. Transcriptome data from monocytes confirmed this possibility, and revealed a significantly high expression level in HBV-related ACLF patients (*P* value = 0.05) (Fig. 2b). Li et al. found that some pro-inflammation molecules, such as TNFα, chemokine (C–C motif) ligand 2 (Ccl2), intercellular adhesion molecule 1 (Icam1), IFNγ, and interleukin 1 beta (IL1β) [28], were highly expressed in the liver of MSP-treated mice, indicating a possible pro-inflammatory effect of MST1L. Therefore, more copies of the MST1L gene likely increase its expression in HBV-related ACLF patients, and may further enhance the intensity of hepatitis inflammation.

The second top associate was a deletion CNV containing a long non-coding RNA gene of HCG4B, and was enriched in the AsCs population. Transcriptome data indicated that the expression of HCG4B was positively correlated with HLA-A, which may be regulated by competing with the sponging microRNAs. Chen et al. also identified a similar expression relationship (r = 0.45, *P* value = 1e−3), and predicted the potential sponging microRNAs [29]. In our study, miR-6823-5p was predicted to be the most likely candidate that could both bind to the HCG4B and 3′UTR of HLA-A (Additional file 7). As one of the important components of the major histocompatibility complex class I (MHC I), HLA-A can influence the CD8+ T-cell response to infected hepatocytes [30] and the levels of cytokine production that greatly associated with the development of autoimmune inflammation [31]. Low copies of HLA-A and its expression level in AsC controls may alleviate inflammation and reduce the risk of ACLF under unknown situations.

Notably, an associated duplicated segment on 8p23.1, containing a cluster of six β defensin genes (DEFB), also tended to appear in HBV-related ACLF patients. DEFB may act as an effective pro-inflammatory factor and also have a strong antiviral effect [32, 33]. Multiple copies of DEFB can enhance host immune activity. Firstly, DEFB can function as chemokines that modulate immune cell migration properties and the localization of target cells such as monocytes, macrophages, immature dendritic cells (DCs), memory T cells, and mast cells [34–37]. Secondly, DEFB are pro-inflammatory and increase the levels of secreted pro-inflammatory molecules TNF-α and IL-6 levels [38].

Our GWAS results indicated a potential excessive inflammatory response in HBV-related ACLF patients or an alleviated inflammatory response in AsC individuals. An excessive inflammatory response may induce tissue damage and organ failure [39], and the systemic inflammation is a potential major driver of ACLF [40]. The related plasma levels of cytokines such as IL-6, IL-10, G-CSF and GM-CSF were higher in ACLF patients (*P* < 0.05) than that in controls [40], and could be associated with the severity and mortality of ACLF [2]. Other than the intensity of the inflammatory response, inflammation-induced tissue damage also depends on the intrinsic capacity of host organs to endure the inflammatory response (individual difference) [39], which is one of the possible reasons that a certain number of AsC individuals contain the risk CNVs but do not reveal symptoms of ACLF.

There are some limitations in the present study. Firstly, there is still a lack of other cohorts or effective experimental techniques to verify the positive results. Secondly, the transcriptome data is not from the samples of this study, and only reflects gene expression in monocytes. Although it can partly illuminate what HBV-related ACLF patients face, more data in regards to this complex disease should be collected to fully illustrate the true expression pattern among different immune cell subsets. Thirdly, although we have initially observed the possibility of a competitive relationship between HLA-A and HCG4B, the sponging miRNA should be further predicted and validated by further exact methods, perhaps by using small RNA sequencing technology and double luciferase reporter gene experiment. Lastly, it is difficult to assess the true effect of these potential risk CNVs in different populations due to the inherent differences among individuals (such as the capacity of enduring the inflammatory response) or other unknown factors.

## Conclusions

The current study observed significant difference in burden of rare CNVs between HBV-related ACLF patients and AsC controls, and also identified a series of disease associated CNVs. The risk CNVs in ACLF patients may further lead to changes of host immunity. Firstly, fewer copies of leukocyte transendothelial migration related genes in patients likely decrease the host cellular immunity. Secondly, copy number variation of genes such as MST1L, DEFB and HCG4B can potentially enhance the inflammatory response of patients during an HBV infection. Our results confirmed that host CNVs can affect the onset of HBV-related ACLF. Future work should foucus on the influence of gene dosage on related pathology, especially abnormal inflammatory response.

## Supplementary information

**Additional file 1.** Summary characteristics of the participants used in this study.

**Additional file 2.** Basic information of the participants used in this study.

**Additional file 3.** Results of merged CNVs predicted by Birdseye and PennCNV.

**Additional file 4.** Genes locating in the significant lost genomic regions (rare CNVs with the size of 100-200 kb).

**Additional file 5.** Key deleted genes in leukocyte transendothelial migration pathway.

**Additional file 6.** Genes locating in the significant gained genomic regions (rare CNVs with the size of 100-200 kb).

**Additional file 7.** Prediction of potential sponging microRNAs between HCG4B and 3’UTR of HLA-A.

## Data Availability

The human reference genome sequence was the assembly version of NCBI36/hg18, as downloaded from University of California, Santa Cruz (UCSC) Genome Browser [ftp://hgdownload.soe.ucsc.edu/goldenPath/hg18/chromosomes/]. The corresponding gene annotation file of hg18 (the reference gene sets) was also downloaded from the UCSC Genome Browser by querying the assembly version of NCBI36/hg18 [http://genome.ucsc.edu/cgi-bin/hgTables]. Transcriptome data was downloaded from NCBI Gene Expression Omnibus (GEO) profiles database (Accession number: GDS4387).

## Abbreviations

CNV: Copy number variation
HBV: Hepatitis B virus
ACLF: Acute-on-chronic liver failure
AsC: Asymptomatic HBV carrier
GWAS: Genome-wide association study
CHB: Chronic hepatitis B
HLA: Human leucocyte antigen
CNVE: Copy number variation event
HCC: Hepatocellular carcinoma
LOD: Logarithm of odds
RPKM: Reads per Kb transcriptome per million mapped reads
GEO: Gene expression omnibus
KEGG: Kyoto encyclopedia of genes and genomes
TG: Target genes
KO: KEGG orthology
MST1L: Macrophage stimulating 1 like
MSP: Macrophage stimulating protein
MHC I: Major histocompatibility complex class I
DEFB: Beta defensin genes
DC: Dendritic cells
