# Supplementary material for: Copy number gain of pro-inflammatory genes in patients with HBV-related acute-on-chronic liver failure
Source: BMC Med Genomics. 2020 Dec 1;13:180. doi: 10.1186/s12920-020-00835-5 (PMC7709420; doi:10.1186/s12920-020-00835-5)
Supplement: Supplementary file 1 — Additional file 1. Summary characteristics of the participants used in this study. [file 12920_2020_835_MOESM1_ESM.doc]

**Additional file 1 Summary characteristics of the participants used in this study.**

| **Variables** | **HBV-ACLFs(n=389)** | **AsCs(n=391)** |
| --- | --- | --- |
| Genotyping platform | Affymetrix SNP 6.0 | Affymetrix SNP 6.0 |
| Age (years, mean±SD) | 41.78±12.07 | 44.32±8.15 |
| Male, n (%) | 335(86.12) | 321(82.10) |
| BMI (mean±SD) | 22.05±3.09 | 22.97±2.56 |
| HBeAg positive, n (%) | 125(32.13) | 62(15.86) |
| HBsAg positive, n (%) | 389(100) | 391(100) |
| HBV DNA (Log_10_copies/mL, mean±SD) | 6.02±1.77 | 2.07±2.65 |
| ALT (IU/L, mean±SD) | 929.56±803.04 | 28.02±12.34 |
| TBil (μmol/L, mean±SD) | 485.48±177.14 | 14.02±5.05 |
| PT (s, mean±SD) | 31.72±12.56 | 11.96±1.31 |
| INR (mean±SD) | 2.46±0.94 | 0.96±0.10 |
| MELD score (mean±SD) | 27.78±7.87 | - |

The population included 389 HBV-ACLF cases and 391 AsCs controls. AsC, asymptomatic HBV carrier; ACLF, acute-on-chronic liver failure; BMI, body mass index; HBsAg, HBV surface antigen; HBeAg, HBV e antigen; ALT, alanine aminotransferase; TBil, total bilirubin; PT, prothrombin time; INR, international normalised ratio; MELD score, the model for end-stage liver disease score.
