# Supplementary material for: Copy number gain of pro-inflammatory genes in patients with HBV-related acute-on-chronic liver failure
Source: BMC Med Genomics. 2020 Dec 1;13:180. doi: 10.1186/s12920-020-00835-5 (PMC7709420; doi:10.1186/s12920-020-00835-5)
Supplement: Supplementary file 4 — Additional file 4. Genes locating in the significant lost genomic regions (rare CNVs with the size of 100-200 kb). [file 12920_2020_835_MOESM4_ESM.doc]

**Additional file 4** Genes locating in the significant lost genomic regions (rare CNVs with the size of 100-200 kb)

| **Chromosomes** | | **Start** | **End** | **Genes** |
| --- | --- | --- | --- | --- |
| chr1 | 1826787 | | 2005699 | CFAP74;CALML6;TMEM52;LOC105378591;GABRD |
| chr1 | 2030623 | | 2224111 | FAAP20 |
| chr1 | 2406284 | | 2569201 | TNFRSF14;PANK4;FAM213B;HES5;MMEL1 |
| chr1 | 6535777 | | 6769957 | TAS1R1;THAP3;ZBTB48;DNAJC11;PHF13;KLHL21;LINC01672 |
| chr1 | 9524960 | | 9630931 | TMEM201 |
| chr1 | 11744362 | | 11915597 | MTHFR;CLCN6;NPPA;KIAA2013;LOC102724659;RNU5E-1;NPPA-AS1;C1orf167;NPPB |
| chr1 | 12040488 | | 12147938 | TNFRSF8 |
| chr1 | 15611657 | | 15761459 | CTRC;CASP9;CELA2A;CELA2B |
| chr1 | 15868733 | | 16010682 | TMEM82;UQCRHL;PLEKHM2;FBLIM1;SLC25A34 |
| chr1 | 16182690 | | 16387073 | C1orf64;HSPB7;FAM131C;CLCNKB;CLCNKA;EPHA2 |
| chr1 | 17391460 | | 17534583 | MIR3972;PADI1;PADI3 |
| chr1 | 19748612 | | 19912407 | HTR6;MINOS1-NBL1;LOC105378614;MINOS1;NBL1 |
| chr1 | 20759367 | | 20861344 | CDA;PINK1;PINK1-AS;MIR6084;DDOST |
| chr1 | 21699153 | | 21811372 | ALPL |
| chr1 | 21982615 | | 22095311 | LDLRAD2 |
| chr1 | 22743176 | | 22911319 | C1QA;C1QC;C1QB;EPHA8;MIR6127 |
| chr1 | 26366019 | | 26515371 | CEP85;SH3BGRL3;ZNF593;CATSPER4;CNKSR1;LOC101928303 |
| chr1 | 29294475 | | 29465960 | MECR;SRSF4;TMEM200B |
| chr1 | 31901503 | | 32040633 | MIR4254;ADGRB2 |
| chr1 | 32673435 | | 32838460 | ZBTB8B |
| chr1 | 38102047 | | 38229079 | SF3A3 |
| chr1 | 39177138 | | 39323829 | NDUFS5;AKIRIN1 |
| chr1 | 43665805 | | 43787536 | MIR6735;HYI |
| chr1 | 45003550 | | 45144658 | PLK3;BTBD19;RPS8;TCTEX1D4;PTCH2;SNORD55;BEST4;SNORD46;SNORD38A;SNORD38B |
| chr1 | 49581990 | | 49755106 | AGBL4-IT1 |
| chr1 | 53693088 | | 53801035 | DMRTB1 |
| chr1 | 54427110 | | 54543497 | SSBP3-AS1;MRPL37 |
| chr1 | 54771870 | | 54903534 | FAM151A;ACOT11 |
| chr1 | 109845040 | | 110003103 | MIR197;AMIGO1;GPR61;GNAT2;GNAI3;AMPD2 |
| chr1 | 149100117 | | 149266361 | CERS2;FAM63A;ANXA9;SETDB1 |
| chr1 | 152195989 | | 152375125 | JTB;MIR6737;RPS27;SLC39A1;CREB3L4;MIR5698;RAB13 |
| chr1 | 152501488 | | 152626450 | AQP10;ATP8B2;HAX1 |
| chr1 | 154012434 | | 154177609 | KIAA0907;SYT11;RIT1;SCARNA26B;SNORA80E;SCARNA4 |
| chr1 | 154740857 | | 154922932 | TTC24;NAXE;LOC101928177;IQGAP3;BCAN;HAPLN2;GPATCH4;NES |
| chr1 | 159166105 | | 159285422 | TSTD1;F11R;ITLN2;USF1;LOC101928372 |
| chr1 | 178362074 | | 178462703 | FLJ23867;QSOX1 |
| chr1 | 185448592 | | 185644632 | LINC01036 |
| chr1 | 209715694 | | 209893090 | SLC30A1;RD3 |
| chr1 | 222354182 | | 222522225 | FBXO28;MIR320B2;LOC101927164;LOC101927143;DEGS1 |
| chr1 | 226119182 | | 226432269 | WNT9A;MIR5008;ARF1;IBA57-AS1;GJC2;MIR3620;MRPL55;C1orf35;GUK1 |
| chr1 | 226540223 | | 226720818 | HIST3H3;MIR4666A;HIST3H2BB;TRIM11;TRIM17;HIST3H2A;MIR6742 |
| chr1 | 228503997 | | 228633132 | PGBD5 |
| chr1 | 233509959 | | 233634410 | GGPS1 |
| chr1 | 240153799 | | 240264827 | BECN2;MAP1LC3C |
| chr1 | 245131520 | | 245250984 | ZNF695 |
| chr2 | 10143258 | | 10283477 | MIR4261;RRM2;C2orf48 |
| chr2 | 20094710 | | 20201501 | LAPTM4A |
| chr2 | 23919606 | | 24096919 | UBXN2A |
| chr2 | 27538144 | | 27726049 | CCDC121;C2orf16;ZNF512;GCKR;FNDC4 |
| chr2 | 28448746 | | 28645460 | FOSL2;FLJ31356 |
| chr2 | 32020376 | | 32175999 | DPY30 |
| chr2 | 38791313 | | 38949111 | GEMIN6;SRSF7 |
| chr2 | 43189371 | | 43328176 | ZFP36L2;LINC01126 |
| chr2 | 55695426 | | 55796917 | PNPT1 |
| chr2 | 60442217 | | 60603445 | MIR4432 |
| chr2 | 61459005 | | 61582017 | SNORA70B |
| chr2 | 70151656 | | 70329379 | TIA1;LINC01816;PCBP1;C2orf42 |
| chr2 | 73115358 | | 73290908 | RAB11FIP5 |
| chr2 | 74167840 | | 74314523 | MTHFD2;BOLA3-AS1;BOLA3;MOB1A |
| chr2 | 74527114 | | 74644843 | MOGS;AUP1;LOXL3;LBX2;INO80B;TTC31;INO80B-WBP1;TLX2;WBP1;HTRA2;DOK1;MRPL53;LBX2-AS1;PCGF1;CCDC142;DQX1 |
| chr2 | 85621077 | | 85757753 | USP39;VAMP5;C2orf68;RNF181;GGCX;VAMP8;SFTPB;TMEM150A |
| chr2 | 96167631 | | 96276514 | DUSP2;STARD7-AS1;STARD7 |
| chr2 | 100509070 | | 100704941 | PDCL3 |
| chr2 | 104745389 | | 104889443 | POU3F3;LINC01159;LINC01158 |
| chr2 | 121646429 | | 121785889 | TFCP2L1 |
| chr2 | 128673255 | | 128817858 | HS6ST1 |
| chr2 | 177663333 | | 177826398 | MIR4444-1;HNRNPA3 |
| chr2 | 201560481 | | 201677819 | NDUFB3 |
| chr2 | 203800305 | | 203906758 | CYP20A1 |
| chr2 | 218415998 | | 218527844 | MIR6809 |
| chr2 | 219422811 | | 219715658 | WNT10A;LOC100129175;CRYBA2;MIR375;LINC00608;LINC01494;WNT6;FEV;CDK5R2;CFAP65;IHH;MIR3131 |
| chr2 | 219948925 | | 220135241 | ASIC4;LOC100996693;DES;SPEG;GMPPA;CHPF;MIR3132;TMEM198 |
| chr2 | 230492002 | | 230628623 | FBXO36 |
| chr2 | 231165677 | | 231312145 | LINC01907 |
| chr2 | 232039978 | | 232180385 | NMUR1;TEX44;LINC00471 |
| chr2 | 232935743 | | 233091660 | ECEL1;ALPP;ALPPL2;ALPI |
| chr2 | 233801535 | | 233903639 | SCARNA6;SCARNA5;ATG16L1 |
| chr2 | 238089423 | | 238197692 | PRLH;RAB17 |
| chr2 | 238880147 | | 239013734 | TRAF3IP1 |
| chr2 | 240931460 | | 241070701 | GPC1;PP14571;MIR149 |
| chr2 | 241591798 | | 241726730 | MTERF4 |
| chr2 | 242046747 | | 242151315 | BOK-AS1;STK25;MIR3133 |
| chr3 | 8852002 | | 9029865 | RAD18 |
| chr3 | 10096435 | | 10212297 | BRK1;VHL;FANCD2OS |
| chr3 | 10386553 | | 10501438 | MIR885 |
| chr3 | 12621435 | | 12804315 | TMEM40 |
| chr3 | 14110271 | | 14233111 | TMEM43;CHCHD4;XPC;LSM3 |
| chr3 | 37975047 | | 38143499 | PLCD1;MIR26A1;DLEC1;VILL |
| chr3 | 46872877 | | 47007828 | MYL3;PTH1R;CCDC12 |
| chr3 | 47137665 | | 47313365 | KIF9-AS1;KIF9 |
| chr3 | 48561701 | | 48723036 | TMEM89;UCN2;SLC26A6;COL7A1;MIR4793;CELSR3;MIR6824;UQCRC1;NCKIPSD;MIR6823;SNORA94;CELSR3-AS1;MIR711 |
| chr3 | 49029683 | | 49196261 | USP19;NDUFAF3;IMPDH2;MIR425;KLHDC8B;QRICH1;MIR6890;MIR191;QARS;LAMB2;CCDC71 |
| chr3 | 49811469 | | 49964906 | MIR5193;MST1R;UBA7;FAM212A;MON1A;TRAIP;CAMKV |
| chr3 | 50153677 | | 50263913 | SEMA3F;SLC38A3;GNAT1;MIR566;MIR5787 |
| chr3 | 51944739 | | 52095982 | PARP3;RPL29;GPR62;PCBP4;ACY1;ABHD14B;ABHD14A-ACY1;DUSP7;LINC00696;ABHD14A |
| chr3 | 57604228 | | 57767503 | LOC101929159 |
| chr3 | 124885517 | | 125012400 | MYLK-AS2 |
| chr3 | 128090913 | | 128258996 | PLXNA1 |
| chr3 | 129529926 | | 129704232 | DNAJB8;GATA2;DNAJB8-AS1 |
| chr3 | 130721938 | | 130824433 | RHO;PLXND1;H1FOO |
| chr3 | 137316457 | | 137452611 | MSL2 |
| chr3 | 165366854 | | 165561675 | MIR1263 |
| chr3 | 177924441 | | 178108556 | LINC01209 |
| chr3 | 178383545 | | 178547841 | LINC00501 |
| chr3 | 195704981 | | 195831370 | TMEM44-AS1 |
| chr3 | 196949765 | | 197058513 | MUC4 |
| chr4 | 58821 | | 215449 | ZNF718 |
| chr4 | 630536 | | 767734 | ATP5I;MYL5;MFSD7;PCGF3;LOC100129917 |
| chr4 | 946047 | | 1102519 | FGFRL1;IDUA;SLC26A1;RNF212 |
| chr4 | 1549523 | | 1660145 | FAM53A |
| chr4 | 3676082 | | 3855606 | ADRA2C |
| chr4 | 4794938 | | 4918223 | LINC01396;MSX1;LOC101928279 |
| chr4 | 6293102 | | 6440543 | WFS1 |
| chr4 | 6595462 | | 6733152 | LOC105374366;MAN2B2;MRFAP1 |
| chr4 | 7315245 | | 7446205 | MIR4798 |
| chr4 | 8187395 | | 8437007 | SH3TC1;HTRA3 |
| chr4 | 9105392 | | 9263088 | MIR548I2 |
| chr4 | 53919706 | | 54073279 | FIP1L1 |
| chr4 | 57401476 | | 57572997 | NOA1;REST |
| chr4 | 65415515 | | 65599025 | LOC401134 |
| chr4 | 71862097 | | 72059306 | GRSF1 |
| chr4 | 129027131 | | 129203690 | MFSD8;ABHD18 |
| chr4 | 152110101 | | 152264188 | RPS3A;SNORD73A |
| chr5 | 142070 | | 329200 | LRRC14B;PLEKHG4B;HRAT5;SDHA;CCDC127 |
| chr5 | 510224 | | 729886 | MIR4456;LOC100288152;PP7080;SLC9A3;LOC100996325;CEP72 |
| chr5 | 1046843 | | 1212749 | SLC12A7;NKD2;MIR4635 |
| chr5 | 1378767 | | 1540663 | SLC6A3;LINC01511 |
| chr5 | 17439984 | | 17568801 | LOC101929544;LOC102723526 |
| chr5 | 37281347 | | 37458875 | NUP155;LOC105374727 |
| chr5 | 40911636 | | 41040153 | C7 |
| chr5 | 54720107 | | 54863336 | MIR5687 |
| chr5 | 55310859 | | 55421130 | FLJ31104 |
| chr5 | 68406254 | | 68584192 | SLC30A5;CCNB1;CENPH;MRPS36 |
| chr5 | 68775777 | | 68903050 | LOC101928924;OCLN |
| chr5 | 78486696 | | 78677247 | JMY |
| chr5 | 132272685 | | 132433375 | ZCCHC10 |
| chr5 | 138960302 | | 139128209 | CXXC5;PSD2-AS1 |
| chr5 | 141445864 | | 141566791 | NDFIP1 |
| chr5 | 148763461 | | 148899539 | MIR143;CARMN;MIR145 |
| chr5 | 154028576 | | 154164616 | MIR1303 |
| chr5 | 159587673 | | 159700056 | CCNJL |
| chr5 | 169954394 | | 170095982 | LOC105377716 |
| chr5 | 172058799 | | 172165227 | LOC101928093;DUSP1 |
| chr5 | 172357861 | | 172499304 | CREBRF;SNORA74B |
| chr5 | 177443555 | | 177556680 | NHP2;RMND5B;N4BP3 |
| chr5 | 178892961 | | 179056241 | RUFY1;CBY3;LOC105377763;HNRNPH1;C5orf60;LOC101928445 |
| chr5 | 179147811 | | 179289563 | MRNIP;LTC4S;SQSTM1;MGAT4B;LOC100996419;MIR1229;TBC1D9B |
| chr6 | 32080632 | | 32288124 | AGPAT1;AGER;TNXB;PPT2;EGFL8;PPT2-EGFL8;MIR6721;PRRT1;RNF5;CYP21A2;LOC100507547;MIR6833;C4B;ATF6B;FKBPL;GPSM3;PBX2 |
| chr6 | 33750571 | | 33932756 | MLN;MIR3934;LEMD2;UQCC2;IP6K3 |
| chr6 | 35452404 | | 35581775 | RPL10A;MIR7111;FANCE;TEAD3 |
| chr6 | 37140907 | | 37333016 | PIM1 |
| chr6 | 39899419 | | 40019219 | LOC100505635;MOCS1 |
| chr6 | 41772099 | | 41904989 | TOMM6;PRICKLE4;FRS3;PGC |
| chr6 | 42816606 | | 42966883 | GLTSCR1L;RPL7L1;LOC401261;TBCC;C6orf226 |
| chr6 | 44232643 | | 44386308 | SLC29A1;SLC35B2;NFKBIE;TCTE1;LOC101929726;HSP90AB1;CAPN11;TMEM151B;MIR4647 |
| chr6 | 74089059 | | 74267413 | KHDC3L;MB21D1;OOEP;DPPA5;DDX43 |
| chr6 | 90526820 | | 90666439 | CASP8AP2;GJA10 |
| chr6 | 108631910 | | 108766337 | SNX3 |
| chr6 | 116480309 | | 116596243 | COL10A1 |
| chr6 | 150060213 | | 150174345 | LOC645967;PCMT1;NUP43 |
| chr6 | 158243466 | | 158351724 | SYNJ2-IT1 |
| chr6 | 159060717 | | 159161435 | MIR3918;EZR |
| chr6 | 160219495 | | 160359056 | AIRN;MAS1 |
| chr6 | 166706327 | | 166853560 | MIR1913;RPS6KA2-IT1 |
| chr6 | 168137269 | | 168242654 | FRMD1;KIF25;KIF25-AS1 |
| chr7 | 868920 | | 1118239 | MIR339;COX19;ADAP1;CYP2W1;GET4;GPER1;GPR146 |
| chr7 | 2414576 | | 2564729 | LOC101927181;MIR4648;GRIFIN;BRAT1;LFNG |
| chr7 | 5065893 | | 5182791 | RBAKDN |
| chr7 | 5402894 | | 5597790 | MIR589;ACTB;FBXL18;LOC221946 |
| chr7 | 27027905 | | 27181932 | HOXA9;HOXA1;HOXA6;HOXA-AS2;HOXA4;HOXA2;HOXA3;HOTAIRM1;MIR196B;HOXA-AS3;HOXA10-AS;HOXA7;HOXA5 |
| chr7 | 44038386 | | 44161486 | MIR6837;PGAM2;MIR6838;POLD2;POLM;MIR4649;AEBP1;DBNL;LINC00957;MYL7 |
| chr7 | 44780021 | | 44932116 | MIR4657;PURB;PPIA;H2AFV |
| chr7 | 52963068 | | 53135052 | POM121L12 |
| chr7 | 55817912 | | 55944423 | 14-Sep |
| chr7 | 55964142 | | 56108112 | CCT6A;MRPS17;SNORA15;GBAS;PSPH |
| chr7 | 72779400 | | 72901942 | CLDN4;CLDN3;ABHD11;WBSCR27;ABHD11-AS1 |
| chr7 | 75426302 | | 75570011 | MDH2;STYXL1;TMEM120A |
| chr7 | 75797940 | | 75912916 | ZP3;SSC4D |
| chr7 | 97451707 | | 97553024 | OCM2 |
| chr7 | 98722203 | | 98962427 | ARPC1B;CPSF4;BUD31;ARPC1A;PTCD1;PDAP1;TRNAW6;ZNF394;ZNF789;ATP5J2;ATP5J2-PTCD1 |
| chr7 | 99428829 | | 99582799 | CNPY4;ZNF3;MCM7;MIR106B;ZKSCAN1;MBLAC1;AP4M1;MIR93;TAF6;COPS6;MIR25;ZSCAN21 |
| chr7 | 104836851 | | 105036398 | PUS7;RINT1;EFCAB10 |
| chr7 | 139619019 | | 139761016 | SLC37A3 |
| chr7 | 139794371 | | 139943448 | MKRN1 |
| chr7 | 149049511 | | 149210881 | ZNF467;ATP6V0E2-AS1;ZNF862;SSPO;ATP6V0E2 |
| chr7 | 149949734 | | 150127601 | GIMAP1-GIMAP5;GIMAP5;GIMAP2;GIMAP6;GIMAP1 |
| chr7 | 158406977 | | 158522557 | LINC00689 |
| chr8 | 509732 | | 712976 | ERICH1;LOC401442 |
| chr8 | 10538743 | | 10659370 | C8orf74;MIR4286;SOX7 |
| chr8 | 11320134 | | 11612211 | BLK;LINC00208;SNORA99 |
| chr8 | 21946392 | | 22137296 | SFTPC;HR;DMTN;FGF17;REEP4;BMP1;FAM160B2;NUDT18;LGI3 |
| chr8 | 22634229 | | 22877865 | LOC101929237 |
| chr8 | 30622913 | | 30795727 | PPP2CB;UBXN8;GSR |
| chr8 | 33464735 | | 33584581 | DUSP26;RNF122;SNORD13;TTI2 |
| chr8 | 37947916 | | 38117812 | EIF4EBP1;ASH2L |
| chr8 | 49037457 | | 49218318 | UBE2V2 |
| chr8 | 81296253 | | 81439746 | MIR5708 |
| chr8 | 101754817 | | 101879667 | PABPC1;MIR7705 |
| chr8 | 131325580 | | 131447492 | ASAP1-IT1 |
| chr8 | 142181689 | | 142287426 | DENND3 |
| chr8 | 142352747 | | 142534149 | LINC01300;GPR20;PTP4A3 |
| chr8 | 142701977 | | 142881346 | MIR1302-7 |
| chr8 | 143163956 | | 143392115 | LINC00051;MIR4472-1 |
| chr8 | 143501471 | | 143747074 | ADGRB1;ARC;LOC101928087 |
| chr8 | 144385752 | | 144510825 | GLI4;MINCR;ZNF696;ZFP41 |
| chr8 | 144888970 | | 145051812 | EPPK1;MIR937;PUF60;NRBP2;SCRIB;MIR6845 |
| chr8 | 145479076 | | 145690967 | MIR6848;SCRT1;MIR6849;TMEM249;CPSF1;DGAT1;MIR939;ADCK5;SLC52A2;LOC101928902;FBXL6;HSF1;TONSL;MIR6893;VPS28;TONSL-AS1;SLC39A4;CYHR1;KIFC2;FOXH1 |
| chr9 | 34143996 | | 34257438 | UBAP1 |
| chr9 | 34503623 | | 34713181 | RPP25L;CNTFR;ARID3C;ENHO;CNTFR-AS1;GALT;DCTN3;SIGMAR1;IL11RA;CCL19;CCL27;LOC730098;CCL21 |
| chr9 | 99663198 | | 99770130 | HEMGN;TRMO |
| chr9 | 99935264 | | 100127858 | TBC1D2;MIR6854 |
| chr9 | 124882861 | | 125013238 | MIR600HG;MIR600 |
| chr9 | 126051516 | | 126208664 | NEK6;LOC100129034 |
| chr9 | 126978288 | | 127162174 | RABEPK;HSPA5;LOC105376271 |
| chr9 | 128330670 | | 128511718 | LMX1B |
| chr9 | 129460273 | | 129710933 | MIR2861;TOR2A;MIR3960;CDK9;PTRH1;SH2D3C;TTC16;MIR3911;CFAP157;ENG;FPGS;ST6GALNAC6;AK1;MIR4672;LOC102723566 |
| chr9 | 129848075 | | 130278198 | PTGES2-AS1;PTGES2;SLC25A25-AS1;SLC25A25;NAIF1;CIZ1;LCN2;MIR3154;MIR199B;C9orf16;DNM1;TRNAR12;GOLGA2;TRUB2;SWI5;COQ4;MIR219B;URM1;CERCAM;MIR219A2 |
| chr9 | 130697873 | | 130877656 | SH3GLB2;MIGA2;DOLK;PHYHD1;NUP188 |
| chr9 | 131016329 | | 131173126 | C9orf106;LOC101929331;LINC01503 |
| chr9 | 131207773 | | 131483652 | LINC00963;C9orf50;ASB6;NTMT1 |
| chr9 | 132497963 | | 132628299 | EXOSC2;PRDM12 |
| chr9 | 132764593 | | 132947580 | FIBCD1 |
| chr9 | 133097899 | | 133199037 | FAM78A;PLPP7 |
| chr9 | 133250747 | | 133457770 | SNORD62A;PRRC2B;SNORD62B;UCK1;POMT1 |
| chr9 | 135184416 | | 135337670 | ADAMTS13;SURF4;SURF6;SNORD24;CACFD1;RPL7A;SNORD36A;MED22;REXO4;STKLD1;SNORD36B;SNORD36C;SURF2;SLC2A6;SURF1 |
| chr9 | 136293009 | | 136494425 | RXRA;MIR4669 |
| chr9 | 137129535 | | 137294199 | LOC401557 |
| chr9 | 137521001 | | 137621450 | LCN1;OBP2A;LINC01502;MRPS2;LOC101928525;PAEP;C9orf116 |
| chr9 | 137956768 | | 138078710 | UBAC1 |
| chr10 | 3862876 | | 4051997 | LOC105376365;MIR6078 |
| chr10 | 13512845 | | 13627898 | BEND7 |
| chr10 | 43160507 | | 43333499 | FXYD4;HNRNPF |
| chr10 | 70546940 | | 70652181 | SUPV3L1;VPS26A |
| chr10 | 70815492 | | 70938631 | TSPAN15;TACR2 |
| chr10 | 73066960 | | 73256591 | C10orf105;VSIR;MIR7152 |
| chr10 | 73725755 | | 73860710 | DNAJB12 |
| chr10 | 80953273 | | 81093385 | SFTPA1;SFTPA2;LOC105378385 |
| chr10 | 87988517 | | 88110804 | MIR346 |
| chr10 | 98504264 | | 98635017 | LOC102723665;MIR607 |
| chr10 | 99027505 | | 99170388 | FRAT2;RRP12;FRAT1 |
| chr10 | 99574351 | | 99700428 | GOLGA7B;LINC00866 |
| chr10 | 102098513 | | 102212947 | OLMALINC |
| chr10 | 102762273 | | 102868801 | SFXN3;KAZALD1 |
| chr10 | 105035343 | | 105161626 | MIR1307;USMG5;PCGF6;TAF5 |
| chr10 | 120811615 | | 120953577 | PRDX3;SFXN4;FAM45A |
| chr10 | 125320490 | | 125510921 | GPR26 |
| chr10 | 131735321 | | 131846067 | LINC00959 |
| chr10 | 133497122 | | 133691364 | BNIP3;PPP2R2D |
| chr10 | 134696616 | | 134918715 | ADGRA1;ADGRA1-AS1;KNDC1;MIR202;UTF1;MIR202HG;VENTX |
| chr11 | 354390 | | 539959 | RNH1;PTDSS2;HRAS;B4GALNT4;PKP3;SIGIRR;ANO9 |
| chr11 | 1822687 | | 2021199 | H19;MIR4298;LINC01150;MRPL23-AS1;TNNT3;MRPL23;SNORD131;LINC01219;MIR675;LSP1;MIR7847;HOTS |
| chr11 | 2267446 | | 2432151 | TSPAN32;CD81;C11orf21;CD81-AS1;TSSC4;TRPM5 |
| chr11 | 2717317 | | 2914858 | KCNQ1DN;KCNQ1-AS1;SLC22A18AS;SLC22A18;PHLDA2;CDKN1C |
| chr11 | 2973028 | | 3100800 | CARS |
| chr11 | 3679463 | | 3808016 | PGAP2 |
| chr11 | 12063247 | | 12217288 | MIR6124 |
| chr11 | 17355181 | | 17480594 | KCNJ11;ABCC8 |
| chr11 | 18381515 | | 18514762 | TSG101;LDHAL6A;LDHC |
| chr11 | 44485591 | | 44598833 | CD82 |
| chr11 | 46254207 | | 46382835 | MDK;DGKZ;CHRM4;MIR4688;CREB3L1 |
| chr11 | 47262467 | | 47411338 | MIR4487;SPI1;SLC39A13;MYBPC3;PSMC3 |
| chr11 | 47476920 | | 47607569 | FAM180B;NDUFS3;C1QTNF4;KBTBD4;PTPMT1 |
| chr11 | 47663548 | | 47799464 | FNBP4 |
| chr11 | 55378180 | | 55517290 | OR5I1;OR10AG1;OR5W2;TRIM51 |
| chr11 | 61229549 | | 61414433 | MYRF;MIR1908;TMEM258;MIR611;FADS2;FEN1;FADS1;DKFZP434K028;MIR6746 |
| chr11 | 61595609 | | 61740232 | SCGB1D1;INCENP;SCGB2A1 |
| chr11 | 62055123 | | 62234898 | INTS5;UQCC3;GANAB;METTL12;TUT1;B3GAT3;MIR6747;EML3;ROM1;MTA2;LRRN4CL;UBXN1;C11orf98;LBHD1;SNORA57;EEF1G;GNG3;BSCL2 |
| chr11 | 64055906 | | 64235262 | SLC22A11;SLC22A12 |
| chr11 | 65027491 | | 65172460 | SSSCA1;MASCRNA;LTBP3;PCNX3;FAM89B;SSSCA1-AS1;EHBP1L1;SCYL1;KCNK7;MIR4690;MAP3K11 |
| chr11 | 65744096 | | 65857236 | KLC2;TMEM151A;CD248;CNIH2;RAB1B;YIF1A |
| chr11 | 66574944 | | 66745524 | RHOD |
| chr11 | 67745740 | | 67909298 | C11orf24 |
| chr11 | 69133252 | | 69394805 | ORAOV1;FGF19;CCND1;FGF4;FGF3 |
| chr11 | 70116801 | | 70306486 | SHANK2-AS1 |
| chr11 | 72630742 | | 72782289 | ARHGEF17;P2RY6 |
| chr11 | 74379732 | | 74504925 | OR2AT4 |
| chr11 | 74781136 | | 74939799 | SNORD15A;KLHL35;SNORD15B;RPS3;GDPD5 |
| chr11 | 119012450 | | 119116143 | LOC102724301;NECTIN1 |
| chr11 | 119386736 | | 119559859 | TRIM29 |
| chr11 | 122375893 | | 122527293 | SNORD14C;SNORD14E;HSPA8;SNORD14D |
| chr11 | 124480150 | | 124674025 | PKNOX2-AS1 |
| chr11 | 125702767 | | 125803552 | ST3GAL4;ST3GAL4-AS1 |
| chr11 | 133195429 | | 133319923 | SPATA19;MIR4697HG;MIR4697 |
| chr12 | 6283801 | | 6638216 | SCNN1A;PLEKHG6;CD27-AS1;CD27;TAPBPL;LTBR;VAMP1;TNFRSF1A;MRPL51;LPAR5;SCARNA11;SCARNA10;NCAPD2;CHD4;GAPDH;NOP2;IFFO1;ACRBP |
| chr12 | 7694913 | | 7810001 | GDF3;DPPA3;CLEC4C |
| chr12 | 7902864 | | 8033905 | SLC2A3 |
| chr12 | 31663220 | | 31782323 | ETFBKMT;AMN1 |
| chr12 | 46408156 | | 46538815 | SLC48A1;RAPGEF3;HDAC7 |
| chr12 | 49734727 | | 49870414 | CSRNP2;TFCP2 |
| chr12 | 51891812 | | 52057614 | ESPL1;MFSD5;SP7;PFDN5;AAAS;C12orf10 |
| chr12 | 54783119 | | 54915706 | SMARCC2;ESYT1;NABP2;RNF41;PA2G4;MYL6;TRNAS11;RPL41;MYL6B;ZC3H10 |
| chr12 | 56145698 | | 56296430 | MBD6;DTX3;MIR6758;DCTN2;ARHGAP9;DDIT3;MIR616;KIF5A;PIP4K2C;MARS |
| chr12 | 97464451 | | 97587545 | SLC25A3;SNORA53;IKBIP |
| chr12 | 100262108 | | 100387038 | ARL1 |
| chr12 | 108435545 | | 108565100 | MVK;MMAB |
| chr12 | 108598764 | | 108727928 | FAM222A-AS1;FAM222A |
| chr12 | 109254872 | | 109441995 | FAM216A;VPS29;GPN3;ANAPC7;ARPC3 |
| chr12 | 110969228 | | 111122498 | MIR6861;TRAFD1 |
| chr12 | 111289644 | | 111449866 | RPL6;PTPN11 |
| chr12 | 119121857 | | 119247220 | SIRT4;PXN-AS1;RNU4-2;RNU4-1;PXN |
| chr12 | 119320186 | | 119499844 | SRSF9;COX6A1;RNF10;NRAV;DYNLL1;GATC;COQ5;TRIAP1 |
| chr13 | 19870722 | | 19994977 | MIR4499 |
| chr13 | 44783066 | | 44908470 | TPT1-AS1;SNORA31;SLC25A30;SLC25A30-AS1;TPT1 |
| chr13 | 49326991 | | 49454276 | SPRYD7 |
| chr13 | 109769067 | | 109870733 | MIR8073 |
| chr13 | 112837017 | | 112965009 | PCID2;PROZ |
| chr13 | 112990159 | | 113145501 | LAMP1;LOC101928841;GRTP1-AS1;GRTP1 |
| chr14 | 23580049 | | 23715876 | IRF9;CPNE6;EMC9;PSME2;PCK2;FITM1;DCAF11;CARMIL3;MIR7703;RNF31;PSME1;NRL |
| chr14 | 35294625 | | 35404868 | SNORA101B |
| chr14 | 38568909 | | 38741586 | TRAPPC6B;PNN;SEC23A;GEMIN2 |
| chr14 | 68457540 | | 68558872 | ACTN1-AS1 |
| chr14 | 73539847 | | 73703837 | ALDH6A1;BBOF1 |
| chr14 | 73794800 | | 73903081 | VRTN;ABCD4 |
| chr14 | 76488136 | | 76588467 | IRF2BPL;LINC01629 |
| chr14 | 77199655 | | 77381492 | SLIRP;C14orf178;SNW1;ALKBH1 |
| chr14 | 90572315 | | 90678747 | SNORA11B |
| chr14 | 99168601 | | 99349154 | CYP46A1 |
| chr14 | 99711057 | | 99878453 | SLC25A29;SLC25A47;MIR6764;YY1;MIR345 |
| chr14 | 100094187 | | 100341229 | LINC00523;DLK1 |
| chr14 | 101519630 | | 101714928 | HSP90AA1 |
| chr14 | 101835236 | | 101951777 | ZNF839;CINP;TRNAI15 |
| chr14 | 102047629 | | 102203138 | MIR4309 |
| chr14 | 103097310 | | 103211965 | APOPT1 |
| chr15 | 21901738 | | 22065565 | PWRN2 |
| chr15 | 38713331 | | 38823740 | RAD51-AS1;RAD51 |
| chr15 | 39665500 | | 39816266 | MIR626 |
| chr15 | 40527295 | | 40721923 | HAUS2;LRRC57;SNAP23 |
| chr15 | 41811782 | | 41940694 | ELL3;HYPK;PDIA3;SERINC4;MFAP1;MIR1282;SERF2-C15ORF63;SERF2 |
| chr15 | 53455706 | | 53560064 | C15orf65 |
| chr15 | 64035943 | | 64145569 | MIR4311 |
| chr15 | 64777477 | | 64883712 | SMAD6 |
| chr15 | 68081842 | | 68224387 | MIR629;TLE3 |
| chr15 | 70074162 | | 70221213 | SENP8 |
| chr15 | 72834479 | | 72940997 | CPLX3;MIR6882;LMAN1L;ULK3;MIR4513;CSK |
| chr15 | 73159137 | | 73331887 | LOC105376731;C15orf39 |
| chr15 | 84033468 | | 84140970 | MIR1276;LOC101929679;KLHL25 |
| chr15 | 88519822 | | 88622002 | SEMA4B;CIB1;GDPGP1;NGRN |
| chr15 | 91343984 | | 91469084 | RGMA |
| chr16 | 1677834 | | 1792776 | NUBP2;NME3;SPSB3;IGFALS;MAPK8IP3;MRPS34;EME2;MIR3177 |
| chr16 | 2495839 | | 2694120 | FLJ42627;CEMP1;ATP6V0C;MIR3178;PDPK1;ERVK13-1;AMDHD2 |
| chr16 | 2909092 | | 3074266 | PKMYT1;PAQR4;THOC6;BICDL2;KREMEN2;LOC101929613;HCFC1R1;CLDN9;CLDN6;LOC100128770;LINC00514;TNFRSF12A;MMP25-AS1;MMP25;IL32 |
| chr16 | 12699130 | | 12822756 | MIR4718 |
| chr16 | 14539432 | | 14688155 | BFAR |
| chr16 | 15814896 | | 15963835 | FOPNL |
| chr16 | 18877656 | | 19046150 | COQ7;TMC7;LOC102723385;ITPRIPL2 |
| chr16 | 21513553 | | 21647775 | IGSF6;METTL9 |
| chr16 | 24350924 | | 24497179 | RBBP6 |
| chr16 | 28795446 | | 28933479 | ATP2A1-AS1;CD19;LAT;RABEP2;NFATC2IP;MIR4517;ATP2A1;SPNS1 |
| chr16 | 56547333 | | 56649445 | TEPP;USB1;MMP15;ZNF319 |
| chr16 | 57141605 | | 57261763 | SNORA50A |
| chr16 | 65573132 | | 65896636 | CES4A;LRRC29;TMEM208;TRADD;KIAA0895L;E2F4;MIR328;HSF4;C16orf70;B3GNT9;ELMO3;FBXL8;NOL3;EXOC3L1;PLEKHG4;SLC9A5;FHOD1 |
| chr16 | 68139413 | | 68270246 | MIR1538 |
| chr16 | 68303893 | | 68445586 | NOB1 |
| chr16 | 69244268 | | 69399598 | VAC14-AS1;MTSS1L;VAC14;TRNAG16;TRNAG6;TRNAG5;TRNAG20 |
| chr16 | 70358443 | | 70516253 | ATXN1L;ZNF821 |
| chr16 | 73132305 | | 73258861 | RFWD3 |
| chr16 | 73793655 | | 73897376 | CTRB2;CTRB1;BCAR1;LOC100506281 |
| chr16 | 79516988 | | 79673188 | CENPN;ATMIN;C16orf46;CMC2 |
| chr16 | 83552944 | | 83732736 | ZDHHC7;KIAA0513;FAM92B |
| chr16 | 83886205 | | 83986479 | MIR5093 |
| chr16 | 84469023 | | 84596385 | MIR6774;IRF8 |
| chr16 | 86609062 | | 86722711 | LOC400553 |
| chr16 | 86878363 | | 87044876 | ZNF469 |
| chr16 | 87271607 | | 87465349 | LOC100289580;LOC339059;CDT1;CTU2;RNF166;MIR4722;PIEZO1;APRT;PABPN1L;GALNS;TRAPPC2L |
| chr16 | 88325128 | | 88494668 | SPIRE2;FANCA |
| chr17 | 1039328 | | 1188905 | BHLHA9;TUSC5 |
| chr17 | 1371698 | | 1570425 | RILP;SCARF1;SLC43A2;MIR22HG;PRPF8;MIR22;TLCD2 |
| chr17 | 3730771 | | 3833861 | ATP2A3;P2RX1 |
| chr17 | 5025798 | | 5182791 | SCIMP;LOC100130950 |
| chr17 | 5225204 | | 5342468 | DERL2;RPAIN;DHX33;LOC105371506;C1QBP;NUP88;MIS12 |
| chr17 | 6249890 | | 6371909 | FAM64A;AIPL1 |
| chr17 | 8109587 | | 8238191 | KRBA2;SLC25A35;RPL26;ODF4;ARHGEF15;LOC100128288;RANGRF |
| chr17 | 16144094 | | 16282352 | CENPV;UBB;TRPV2 |
| chr17 | 17037759 | | 17199582 | PLD6;FLCN;COPS3;NT5M |
| chr17 | 17262777 | | 17401399 | MED9;RASD1 |
| chr17 | 17520586 | | 17690463 | MIR33B;MIR6777;RAI1;RAI1-AS1;SMCR5;SREBF1 |
| chr17 | 18014321 | | 18134091 | LLGL1;FLII;MIEF2;ALKBH5 |
| chr17 | 19700516 | | 19857964 | TRNAG26;AKAP10 |
| chr17 | 21090473 | | 21239691 | MAP2K3 |
| chr17 | 24405657 | | 24584663 | TIAF1;MYO18A |
| chr17 | 27651258 | | 27809879 | C17orf75;ZNF207;MIR632 |
| chr17 | 35540614 | | 35710677 | MIR6866;CASC3;WIPF2;RAPGEFL1;MIR6867 |
| chr17 | 37362793 | | 37604501 | DNAJC7;KAT2A;CNP;DHX58;ZNF385C;HSPB9;NKIRAS2;HCRT;RAB5C;KCNH4;GHDC |
| chr17 | 37771683 | | 37911892 | MIR548AT;PTRF |
| chr17 | 38328835 | | 38434583 | AARSD1;PTGES3L;IFI35;RPL27;PTGES3L-AARSD1;VAT1;RUNDC1 |
| chr17 | 39481318 | | 39610662 | G6PC3;LOC105371789;C17orf53;HDAC5 |
| chr17 | 40400206 | | 40553119 | DCAKD;MIR6784;NMT1 |
| chr17 | 41173230 | | 41298840 | CRHR1;SPPL2C |
| chr17 | 41569943 | | 41719991 | KANSL1-AS1 |
| chr17 | 42921890 | | 43078270 | NPEPPS |
| chr17 | 44846582 | | 44992369 | MIR6165;LINC02075;NGFR |
| chr17 | 45502679 | | 45648902 | LINC01969;SAMD14;COL1A1;PDK2;SGCA;PPP1R9B |
| chr17 | 45956380 | | 46151686 | CACNA1G-AS1;LOC105371824;EPN3;SPATA20;CACNA1G;ANKRD40;ABCC3 |
| chr17 | 46229048 | | 46336583 | TOB1-AS1;WFIKKN2;TOB1 |
| chr17 | 54643033 | | 54762977 | GDPD1 |
| chr17 | 62713554 | | 62872379 | PSMD12 |
| chr17 | 63149569 | | 63261952 | SNORA38B |
| chr17 | 63717430 | | 63838180 | SLC16A6 |
| chr17 | 68680978 | | 68782184 | C17orf80;FAM104A;COG1;CPSF4L |
| chr17 | 71958946 | | 72101802 | RHBDF2;SNORD1C;CYGB;PRCD;ST6GALNAC2;SNORD1A;SNHG16;AANAT;SNORD1B |
| chr17 | 72407940 | | 72556821 | LOC105371899 |
| chr17 | 72872203 | | 73040909 | MIR4316 |
| chr17 | 73657612 | | 73765232 | TK1;SYNGR2;AFMID;TMEM235;BIRC5 |
| chr17 | 74333763 | | 74491005 | LGALS3BP;CEP295NL;TIMP2 |
| chr17 | 74510115 | | 74676812 | ENGASE;C1QTNF1;C1QTNF1-AS1 |
| chr17 | 75923703 | | 76109564 | MIR4730;NPTX1;LOC100294362;ENDOV |
| chr17 | 76648559 | | 76796220 | MIR3065;MIR338;AATK;MIR657;AATK-AS1;MIR1250 |
| chr17 | 77502621 | | 77680388 | GPS1;DCXR;FASN;CENPX;ASPSCR1;LRRC45;RAC3;DUS1L;RFNG;NOTUM;SNORD134 |
| chr17 | 77987819 | | 78136439 | NARF;C17orf62;TRNAM14 |
| chr17 | 78272237 | | 78470368 | ZNF750;FN3K |
| chr18 | 17376922 | | 17568369 | MIR320C1;ABHD3;SNRPD1 |
| chr18 | 41807008 | | 42002359 | TRNAK4;HAUS1;PSTPIP2;ATP5A1 |
| chr18 | 42818160 | | 42949515 | HDHD2 |
| chr18 | 44633244 | | 44783125 | SMAD7 |
| chr18 | 53431207 | | 53554370 | LOC100505549 |
| chr19 | 2301208 | | 2460355 | LMNB2;MIR7108;LINC01775;TIMM13;TMPRSS9;GADD45B |
| chr19 | 2595471 | | 2963883 | SLC39A3;SGTA;DIRAS1;ZNF555;ZNF554;ZNF556;THOP1;ZNF77;ZNF57;TLE6 |
| chr19 | 4293353 | | 4524638 | CHAF1A;HDGFRP2;MIR4746;SH3GL1;PLIN4;UBXN6;MPND;SEMA6B;LRG1;PLIN5 |
| chr19 | 4808622 | | 5032481 | UHRF1;MIR4747;ARRDC5 |
| chr19 | 5758414 | | 5934522 | FUT5;NDUFA11;CAPS;VMAC;NRTN;FUT3;FUT6;RANBP3;LOC101928844 |
| chr19 | 6431965 | | 6605959 | TUBB4A;CD70;TNFSF9 |
| chr19 | 6717213 | | 6849433 | VAV1 |
| chr19 | 7357300 | | 7525239 | LOC100128573;ARHGEF18;C19orf45;ZNF358;PEX11G;MCOLN1 |
| chr19 | 7825370 | | 8069562 | TGFBR3L;TIMM44;LRRC8E;SNAPC2;MAP2K7;PRR36;CTXN1;ELAVL1;CCL25 |
| chr19 | 8098290 | | 8236622 | CERS4 |
| chr19 | 8387765 | | 8613884 | ADAMTS10;MYO1F;ZNF414;PRAM1;HNRNPM |
| chr19 | 8968432 | | 9147595 | OR1M1;ZNF317;OR7G2;OR7G1;OR7G3 |
| chr19 | 11023121 | | 11140255 | MIR6886;SPC24;LDLR |
| chr19 | 11341638 | | 11457297 | PRKCSH;RGL3;ELAVL3;SWSAP1;EPOR;CCDC151 |
| chr19 | 13644326 | | 13786410 | C19orf53;CCDC130;MRI1 |
| chr19 | 14486790 | | 14643486 | NDUFB7;SNORA104;CLEC17A;MIR639;TECR |
| chr19 | 15186257 | | 15347059 | EPHX3;BRD4 |
| chr19 | 16053625 | | 16319436 | HSH2D;CIB3;RAB8A;FAM32A;AP1M1;KLF2 |
| chr19 | 16393443 | | 16559052 | CALR3;C19orf44;SLC35E1;CHERP |
| chr19 | 20992242 | | 21095612 | ZNF430 |
| chr19 | 38124296 | | 38308096 | RHPN2;FAAP24 |
| chr19 | 38351101 | | 38490177 | SLC7A10;CEBPA;LRP3;CEBPA-AS1;TRNAT4 |
| chr19 | 39511999 | | 39696459 | UBA2;GPI;PDCD2L;WTIP |
| chr19 | 41825125 | | 41930856 | LINC01534;ZNF567 |
| chr19 | 44079556 | | 44259756 | SARS2;MRPS12;NFKBIB;FBXO27;FBXO17;CCER2 |
| chr19 | 44285672 | | 44584110 | NCCRP1;IFNL3;PAK4;SYCN;LRFN1;IFNL1;SAMD4B;IFNL2;GMFG;PAF1;MED29 |
| chr19 | 48603560 | | 48736388 | ETHE1;ZNF575;LYPD3;PHLDB3 |
| chr19 | 55598980 | | 55728083 | MYBPC2;SPIB;FAM71E1;EMC10;JOSD2;ASPDH |
| chr19 | 56075992 | | 56177006 | KLK4;KLK6;KLK5 |
| chr19 | 56227769 | | 56351571 | SIGLEC9;KLK14;SIGLEC7;KLK13;CTU1 |
| chr19 | 57591368 | | 57693111 | ZNF528;ZNF534 |
| chr19 | 60149819 | | 60266719 | NLRP2;GP6 |
| chr19 | 61135191 | | 61248580 | NLRP8 |
| chr20 | 3084703 | | 3185893 | LZTS3;ITPA;SLC4A11;DDRGK1 |
| chr20 | 3738831 | | 3919803 | MIR103B2;MIR103A2;MAVS;PANK2;AP5S1 |
| chr20 | 5441623 | | 5558737 | GPCPD1 |
| chr20 | 14792049 | | 14957054 | MACROD2-AS1 |
| chr20 | 25139773 | | 25399586 | PYGB;ABHD12;GINS1 |
| chr20 | 28034013 | | 28165872 | LINC01597 |
| chr20 | 33076973 | | 33256820 | PROCR;EDEM2 |
| chr20 | 33503644 | | 33627140 | MIR1289-1;C20orf173;CEP250;ERGIC3 |
| chr20 | 39381057 | | 39492426 | EMILIN3;LPIN3 |
| chr20 | 41652333 | | 41782996 | IFT52;MYBL2 |
| chr20 | 45369692 | | 45486904 | LOC101927377;LOC100131496 |
| chr20 | 46402457 | | 46569823 | LINC00494 |
| chr20 | 47596494 | | 47785605 | B4GALT5 |
| chr20 | 48025795 | | 48215941 | TMEM189-UBE2V1;UBE2V1;TRERNA1;TMEM189;SNAI1 |
| chr20 | 48689231 | | 48890676 | PARD6B;LOC100506175 |
| chr20 | 54610972 | | 54718233 | TFAP2C |
| chr20 | 56760036 | | 56917480 | GNAS-AS1;MIR298;MIR296;LOC105372695;LOC101927932 |
| chr20 | 60157383 | | 60413803 | OSBPL2;HRH3;MTG2;ADRM1;LAMA5;RPS21;MIR4758;LAMA5-AS1 |
| chr20 | 61019647 | | 61209560 | BHLHE23;LINC01056;HAR1A;LINC00029;LINC01749;GID8;HAR1B;SLC17A9 |
| chr20 | 61734769 | | 61858539 | LIME1;ZGPAT;STMN3;RTEL1;RTEL1-TNFRSF6B;TNFRSF6B;ARFRP1;SLC2A4RG |
| chr20 | 62034232 | | 62217111 | LINC00176;MIR6813;NPBWR2;ZNF512B;MIR1914;MIR647;OPRL1;LKAAEAR1;SAMD10;UCKL1;TCEA2;RGS19;PRPF6;SOX18;UCKL1-AS1 |
| chr21 | 31759249 | | 31900780 | LOC150051 |
| chr21 | 36403825 | | 36509429 | CBR3-AS1;CBR3 |
| chr21 | 36595251 | | 36721694 | CHAF1B;MORC3 |
| chr21 | 42232238 | | 42366126 | SNORA91;ZNF295-AS1;ZBTB21 |
| chr21 | 42760054 | | 42891148 | RSPH1;LOC101930094;SLC37A1 |
| chr21 | 43612690 | | 43755376 | LINC00313;SIK1;LINC00319 |
| chr21 | 43931713 | | 44081789 | AATBC;RRP1;CSTB;PDXK |
| chr21 | 45093924 | | 45291789 | FAM207A;ITGB2-AS1;LINC01547;LINC00163;PICSAR;ITGB2;PTTG1IP |
| chr21 | 45613199 | | 45824932 | COL18A1-AS1;MIR6815;COL18A1-AS2;SLC19A1;COL18A1 |
| chr21 | 46139330 | | 46260755 | COL6A1;LOC101928796 |
| chr21 | 46366648 | | 46560163 | FTCD;MCM3AP;LSS;SPATC1L;MCM3AP-AS1;YBEY |
| chr22 | 16438748 | | 16569296 | LOC101929372;ATP6V1E1 |
| chr22 | 16671271 | | 16868132 | MIR648 |
| chr22 | 18395016 | | 18665127 | ZDHHC8;RANBP1;MIR6816;MIR185;MIR3618;MIR1306;CCDC188;DGCR8;TRMT2A;MIR1286;LINC00896;RTN4R;LOC284865 |
| chr22 | 21292474 | | 21432759 | GGTLC2;IGLV3-19;IGLV3-21;IGLV2-18;IGLV3-25;IGLV3-16;IGLV2-14;IGLV3-22;IGLV2-23;IGLV3-27 |
| chr22 | 22432408 | | 22536833 | DERL3;C22orf15;MMP11;CHCHD10;SMARCB1 |
| chr22 | 22832489 | | 23016498 | SUSD2;GGT5 |
| chr22 | 23603546 | | 23711045 | TMEM211 |
| chr22 | 28219146 | | 28347000 | NIPSNAP1;THOC5 |
| chr22 | 30364475 | | 30562539 | PRR14L |
| chr22 | 33791338 | | 33897413 | ISX |
| chr22 | 34158598 | | 34352292 | MB;RASD2 |
| chr22 | 35897773 | | 36044817 | RAC2;SSTR3;C1QTNF6;CYTH4 |
| chr22 | 36318134 | | 36548583 | LGALS1;LOC101927051;NOL12;GGA1;PDXP;SH3BP1;GCAT;TRIOBP;H1F0 |
| chr22 | 36572759 | | 36692417 | C22orf23;EIF3L;MIR659;MICALL1 |
| chr22 | 37311696 | | 37466069 | CBY1;TOMM22;JOSD1;GTPBP1 |
| chr22 | 40616201 | | 40717096 | MIR33A;SHISA8;MIR378I;LINC00634;TNFRSF13C;CENPM |
| chr22 | 41089285 | | 41240713 | LINC01315;NFAM1 |
| chr22 | 43346431 | | 43460030 | LINC00229 |
| chr22 | 43653782 | | 43787800 | PHF21B |
| chr22 | 43835746 | | 43950310 | NUP50-AS1 |
| chr22 | 45035925 | | 45229194 | GTSE1-AS1;TTC38;TRMU;GTSE1 |
| chr22 | 47245695 | | 47433153 | LOC284933 |
| chr22 | 49249097 | | 49349366 | SYCE3;MIOX;ODF3B;KLHDC7B;SCO2;TYMP;ADM2;NCAPH2;LMF2 |

Note: The reference genome is the human genome version hg18.
