# Supplementary material for: Copy number gain of pro-inflammatory genes in patients with HBV-related acute-on-chronic liver failure
Source: BMC Med Genomics. 2020 Dec 1;13:180. doi: 10.1186/s12920-020-00835-5 (PMC7709420; doi:10.1186/s12920-020-00835-5)
Supplement: Supplementary file 5 — Additional file 5. Key deleted genes in leukocyte transendothelial migration pathway. [file 12920_2020_835_MOESM5_ESM.doc]

**Additional file 5** Key deleted genes in leukocyte transendothelial migration pathway

| **Node name** | **Node or gene production description** | **KEGG ID** | **Genes in the node** |
| --- | --- | --- | --- |
| CAMs | cell adhesion molecule | K06088;K06087 | CLDN3, CLDN4, CLDN6, CLDN9, OCLN |
| ITGB2 | integrin subunit beta 2 | K06464 | ITGB2 |
| SHP-2 | protein tyrosine phosphatase non-receptor type 11 | K07293 | PTPN11 |
| Vav | vav guanine nucleotide exchange factors | K05703 | VAV1, VAV2 |
| Actin | actin beta | K05692 | ACTB |
| MLC | myosin light chain | K12754;K12753 | MYL5, MYL7 |
| ERM | Villin 2 (ezrin) | K08007 | VIL2 |
| P130Cas | breast cancer anti-estrogen resistance 1 | K05726 | BCAR1 |
| JAM | junctional adhesion molecule 1 | K06089 | F11R |
| Rac2 | Ras-related C3 botulinum toxin substrate 2 | K07860 | RAC2 |
| Gi | guanine nucleotide-binding protein G(i) subunit alpha | K04630 | GNAI |
| EPAC | Rap guanine nucleotide exchange factor 4 | K08014 | RAPGEF4 |
