# Supplementary material for: Copy number gain of pro-inflammatory genes in patients with HBV-related acute-on-chronic liver failure
Source: BMC Med Genomics. 2020 Dec 1;13:180. doi: 10.1186/s12920-020-00835-5 (PMC7709420; doi:10.1186/s12920-020-00835-5)
Supplement: Supplementary file 6 — Additional file 6. Genes locating in the significant gained genomic regions (rare CNVs with the size of 100-200 kb). [file 12920_2020_835_MOESM6_ESM.doc]

**Additional file 6** Genes locating in the significant gained genomic regions (rare CNVs with the size of 100-200 kb)

| **Chromosomes** | **Start** | **End** | **Genes** |
| --- | --- | --- | --- |
| chr1 | 12832564 | 12938082 | PRAMEF10;PRAMEF2;PRAMEF6;PRAMEF7;PRAMEF4 |
| chr1 | 47131119 | 47279745 | CYP4A11 |
| chr1 | 100622778 | 100820318 | GPR88 |
| chr1 | 144337063 | 144503421 | CD160;PDZK1 |
| chr1 | 157919215 | 158102297 | DUSP23;C1orf204;SLAMF8;VSIG8;CRP;FCRL6 |
| chr1 | 169477015 | 169586632 | FMO4;FMO1 |
| chr1 | 171984851 | 172088063 | CENPL |
| chr1 | 225187722 | 225375039 | COQ8A |
| chr1 | 247044430 | 247191011 | ZNF672;TRNAL19;TRNAE24;ZNF692;SH3BP5L;PGBD2;MIR3124 |
| chr2 | 2784 | 178421 | FAM110C |
| chr2 | 32638062 | 32768873 | MIR4765 |
| chr2 | 56137774 | 56248405 | LINC01813 |
| chr2 | 95556954 | 95734561 | TRIM43 |
| chr2 | 95832268 | 95946534 | LINC00342 |
| chr2 | 111718360 | 111912704 | MIR4435-2 |
| chr2 | 136105253 | 136225930 | MIR128-1 |
| chr2 | 138572865 | 138675522 | LINC01832 |
| chr2 | 232912603 | 233034168 | ALPPL2;ALPP;ALPI |
| chr3 | 18399384 | 18598662 | SATB1-AS1 |
| chr3 | 165348207 | 165521800 | MIR1263 |
| chr3 | 166110181 | 166308018 | SI |
| chr3 | 176423433 | 176575026 | NAALADL2-AS2;MIR4789 |
| chr3 | 199233295 | 199380515 | LMLN-AS1 |
| chr4 | 2589129 | 2715532 | FAM193A |
| chr4 | 6573539 | 6675473 | MAN2B2 |
| chr4 | 8979788 | 9095185 | DEFB131 |
| chr4 | 48599323 | 48788543 | CWH43 |
| chr4 | 70466094 | 70592101 | UGT2A1;UGT2A2 |
| chr4 | 70927899 | 71056529 | HTN1;HTN3 |
| chr4 | 137004430 | 137191227 | LINC00613 |
| chr5 | 26906541 | 27096600 | CDH9 |
| chr5 | 28270279 | 28393273 | LINC02103 |
| chr5 | 36281189 | 36409786 | RANBP3L |
| chr5 | 175456803 | 175583090 | LOC100507387 |
| chr6 | 68807361 | 68926407 | LOC102723883 |
| chr6 | 150165976 | 150281023 | RAET1E;LRP11 |
| chr7 | 22383400 | 22508292 | STEAP1B |
| chr7 | 22575496 | 22679742 | LOC401312 |
| chr7 | 22823144 | 22948053 | SNORD93 |
| chr7 | 29640265 | 29750238 | MIR550A3;LOC646762 |
| chr7 | 93136911 | 93251419 | MIR4652 |
| chr7 | 104805732 | 104970246 | PUS7 |
| chr7 | 123634512 | 123789366 | LOC101928211 |
| chr8 | 6863552 | 6963604 | DEFA5 |
| chr8 | 56720970 | 56885304 | TMEM68 |
| chr8 | 75605605 | 75766232 | MIR5681B;MIR5681A |
| chr8 | 83886445 | 84057292 | LOC101927141 |
| chr8 | 93786269 | 93975352 | FLJ46284 |
| chr9 | 91857 | 279431 | C9orf66;CBWD1;FOXD4 |
| chr10 | 35096045 | 35227595 | PARD3-AS1 |
| chr10 | 57710257 | 57909670 | ZWINT |
| chr10 | 58596001 | 58765460 | MIR3924 |
| chr10 | 66290913 | 66476769 | LOC101928887 |
| chr10 | 135160323 | 135356694 | SYCE1;CYP2E1;FRG2B |
| chr11 | 55609746 | 55785098 | OR8J3;OR5T2;OR5J2;OR8K5;OR8H2;OR8I2;OR8H3;OR5T3 |
| chr11 | 57809764 | 57932847 | OR5B17;OR5B3 |
| chr11 | 107127988 | 107316487 | SLC35F2 |
| chr12 | 225811 | 333730 | LOC102723544 |
| chr12 | 14584281 | 14706173 | PLBD1-AS1 |
| chr12 | 89830322 | 89987152 | EPYC;CCER1;LINC00615;KERA |
| chr12 | 132111029 | 132288262 | ZNF891;LOC101928597;ZNF10;ZNF84;ZNF140 |
| chr13 | 49061822 | 49181820 | ARL11;EBPL |
| chr13 | 57058080 | 57210023 | PCDH17 |
| chr13 | 63115883 | 63271229 | LINC00395 |
| chr13 | 66284302 | 66412501 | PCDH9-AS2 |
| chr13 | 68283613 | 68404850 | LINC00550 |
| chr13 | 83209220 | 83375462 | SLITRK1 |
| chr13 | 87165385 | 87300073 | LINC00397 |
| chr14 | 21704333 | 21816094 | TRAV38-1;TRAV30;TRAV26-2;TRAV35;TRAV36DV7;TRAV34 |
| chr14 | 106030687 | 106177853 | IGHV4-59;IGHV3-53;IGHV5-51;IGHV3-48;IGHV4-61;IGHV1-58;IGHV3-49;IGHV1-45;IGHV1-46 |
| chr15 | 41672410 | 41783180 | CATSPER2;STRC;CKMT1A |
| chr16 | 8818255 | 8934942 | CARHSP1 |
| chr16 | 18839092 | 18989602 | TMC7;LOC102723385 |
| chr16 | 20991613 | 21124562 | TMEM159 |
| chr16 | 27084065 | 27266695 | KDM8;NSMCE1;FLJ21408 |
| chr16 | 27301766 | 27476553 | IL21R;IL21R-AS1;GTF3C1 |
| chr17 | 21272823 | 21459693 | C17orf51 |
| chr17 | 41569943 | 41719991 | KANSL1-AS1 |
| chr19 | 19885518 | 20036143 | ZNF682 |
| chr19 | 20213206 | 20375423 | MIR1270 |
| chr19 | 44389707 | 44584110 | IFNL3;IFNL1;LRFN1;IFNL2;PAF1;MED29;SAMD4B;GMFG |
| chr19 | 51330710 | 51435841 | LOC93429;IGFL2;IGFL2-AS1;IGFL1 |
| chr19 | 51979347 | 52113465 | AP2S1;SNAR-E |
| chr20 | 45899304 | 46094908 | LINC01522;LINC01523 |
| chr21 | 22342803 | 22469045 | LINC00308 |
| chr21 | 30748734 | 30879179 | KRTAP19-3;KRTAP19-1;KRTAP19-2;KRTAP19-4;KRTAP19-6;KRTAP19-7;KRTAP19-5 |
| chr22 | 17256428 | 17386125 | PRODH;DGCR6 |
| chr22 | 48252141 | 48420991 | MIR3667 |

Note: The reference genome is the human genome version hg18.
