# Supplementary material for: Copy number gain of pro-inflammatory genes in patients with HBV-related acute-on-chronic liver failure
Source: BMC Med Genomics. 2020 Dec 1;13:180. doi: 10.1186/s12920-020-00835-5 (PMC7709420; doi:10.1186/s12920-020-00835-5)
Supplement: Supplementary file 7 — Additional file 7. Prediction of potential sponging microRNAs between HCG4B and 3’UTR of HLA-A. [file 12920_2020_835_MOESM7_ESM.doc]

**Additional file 7** Prediction of potential sponging microRNAs between HCG4B and 3’UTR of HLA-A

| TargetScan | | | | | | | | BLAST score |
| --- | --- | --- | --- | --- | --- | --- | --- | --- |
| Micro RNA | Target 1 | | | Target 2 | | | seed |  |
|  | gene | start | end | gene | start | end |  |  |
| miR-6823-5p | HCG4B | 717 | 724 | HLA-A 3'UTR | 66 | 73 | 8mer | 34.7 |
| miR-1249-5p/6797-5p | HCG4B | 148 | 154 | HLA-A 3'UTR | 192 | 198 | 7mer | 32.8 |
| miR-450a-2-3p | HCG4B | 729 | 735 | HLA-A 3'UTR | 196 | 202 | 7mer | 32.8 |
| miR-4524a-3p | HCG4B | 828 | 835 | HLA-A 3'UTR | 248 | 255 | 8mer | 32.8 |
| miR-510-5p | HCG4B | 642 | 649 | HLA-A 3'UTR | 307 | 314 | 8mer | 32.8 |
| miR-6825-5p | HCG4B | 727 | 734 | HLA-A 3'UTR | 194 | 201 | 8mer | 32.8 |
